# Multi-label machine learning for power forecasting of a grid-connected photovoltaic solar plant over multiple time horizons

**DOI:** 10.1038/s41598-025-20251-y

**Published:** 2025-09-23

**Authors:** Amal A. Hassan, Doaa M. Atia, Hanaa T. El-Madany, Fatma ElGhannam

**Affiliations:** 1 Photovoltaic Cells Department, Electronics Research Institute, Cairo, Egypt; 2 Informatics Research Department, Electronics Research Institute, Cairo, Egypt

**Keywords:** Photovoltaic power plant, Grid-connected, Machine learning, Forecasting, Dataset, Pre-processing, Electrical and electronic engineering, Photovoltaics

## Abstract

Because of solar power’s inherent intermittency and stochastic nature, accurate photovoltaic (PV) generation forecasting is critical for the planning and operation of PV-integrated power systems. Thus, accurate power forecasting becomes vital for maintaining good power dispatch efficiency and power grid operational security. Several PV forecasting methods based on machine learning algorithms (MLAs) have recently emerged. This paper presents machine learning methods for multi-label forecasting of PV and AC power delivered to the grid of a building-applied PV plant. Various algorithms representing multiple groups are evaluated, including linear regression (LR), polynomial regression (PR), neural networks (NN), deep learning (DL), gradient-boosted trees (GBT), random forests (RF), decision trees (DT), k-nearest neighbor (k-NN), and support vector machines (SVM). The models use real-time collected data from sensors over one year for solar irradiance, ambient temperature, wind speed, and cell temperature to predict PV and AC power outputs. Forecast performance over multiple time horizons is validated using four datasets: 24 h, one week, one month, and sudden variations. Models are evaluated based on performance metrics such as absolute error (AE), root mean square error (RMSE), normalized absolute error (NAE), relative error (RE), relative root square error (RRSE), and correlation coefficient (R). Results show that RF, DT, and DL consistently achieved the highest accuracy (*R* ≈ 99.8–100%) with minimal errors (RMSE within 0.014–0.022, AE within 0.008–0.015) across various forecasting scenarios. These models demonstrated strong adaptability and predictive reliability across short-term, medium-term, and long-term forecasts, making them the most effective choices for PV and AC power prediction. The accurate forecasts generated in this study have the potential to aid grid operators in forecasting PV power output variability and planning for integrating intermittent PV power into the grid. Understanding how PV generation will fluctuate given different meteorological conditions allows operators to ensure the consistent integration of this weather-dependent power source. Moreover, multi-label prediction of DC and AC power enables inverter efficiency optimization and grid integration analysis. The average actual and predicted efficiencies of the inverter are 0.96688 and 0.9638, providing valuable insights.

## Introduction

### Background

Renewable energy sources, especially solar, have become a primary focus in recent years due to their ability to be used for extended periods without causing harm to the environment. Emerging energy issues, such as unreliable supply and regional power shortages, could be mitigated by evolving renewable energy technologies. However, the use of diverse but unstable energy sources from renewables is crucial due to the unpredictability of the energy market and the intermittent nature of these alternatives. Figuring out how to smoothly manage fluctuations from renewable energy remains a core challenge. Monitoring energy usage more precisely can boost overall energy system efficiency. Applying energy forecasting can help governments at all levels better develop, implement, and adjust energy policies^1^. Properly incorporating and managing these resources into the existing infrastructure has become a major responsibility for the energy industry, particularly in areas heavily reliant on climate-sensitive energy supplies^1^. Output from these systems fluctuates based on power creation, storage capacity, and demand, endangering the reliability and stability of the overall energy system^2^. Managing and integrating distributed energy resources has created new prospects for developing novel business and market models leveraging the benefits of these technologies. Enabling technologies like blockchain, the Internet of Things, and especially artificial intelligence aim to facilitate these advances. Integrating PV arrays with sophisticated grid management using AI enables perceptive power flow optimization and reduced losses. This opens up opportunities for new business models, encouraging consumers to trade surplus energy and participate in demand response programs^2^. Numerous researchers have extensively researched and advanced the ability to harness renewable energy sources such as solar, wind, and hydropower. It is expected that, in the near future, 100% of energy will come from these sustainable resources. Consequently, renewable technologies are expected to play a vital role in Egypt’s energy landscape, with solar energy being the most predominant renewable resource in the country^3^.

The variability in PV generation poses substantial challenges for managing the current power infrastructure. The fluctuating output from solar panels makes it difficult to match with electricity demand, creating issues in maintaining an immediate supply-demand balance. This becomes increasingly complex and costly as solar penetration grows, affecting decisions related to backup resources, scheduling, storage, and long-term planning. Integrating high levels of solar PV necessitates significant adjustments in power system management and electricity trading^3,4^. Forecasting solar power accurately is essential to addressing these challenges. Various techniques, including numerical weather prediction, image-based analysis, statistical methods, and hybrid neural network approaches, are used to estimate solar irradiance and PV output^4^. Emerging technologies like artificial intelligence (AI) and machine learning (ML) are improving solar forecasting by enhancing accuracy and efficiency. AI and ML help power grid operators make informed decisions, plan operations, and optimize energy market strategies, reducing costs associated with balancing intermittent solar power fluctuations^5,6^. Forecasting models fall into two main categories: indirect and direct. Indirect forecasting predicts solar irradiance using various methods and then estimates PV generation through simulation software^7^. Direct forecasting uses historical data to predict the PV power output. Both methods aim to improve PV power forecasting for grid integration. Forecasts can be categorized by their horizon: very short-term (seconds to minutes), short-term (1 day to several days), medium-term (1 week to 1 month), and long-term (1 month to 1 year). Each type serves different needs, from minute-by-minute fluctuations to seasonal and annual trends, guiding resource planning and investment^2,7^.

### Literature review

Several prior studies developed PV power forecasting models using machine learning techniques. Dan Assouline et al.^8^ applied support vector machine (SVM) models based on the kernel technique to estimate rooftop PV potential across urban Switzerland. In^9^, Stefan Preda et al. focus on hybrid renewable energy power forecasting by applying the SVM algorithm using real data generated from sensors. By processing large datasets, their results validated that big data analysis enhances predictive accuracy for renewable energy resources. Muhammad W. et al.^10^ studied the accuracy, stability, and computational cost of two tree-based models, mainly extra trees (ET), and random forest (RF) for PV power hourly prediction. The authors suggested, as a future study, the development of models based on weather classification, including clear and cloudy days. Also, they suggested the need to explore big data analysis tools for training and installing renewable energy forecast models. Rogério C. Costa^11^ utilized deep learning to predict residential PV system generation. Real data was employed to evaluate the forecast accuracy of long-short-term memory (LSTM), convolutional, and hybrid convolutional-LSTM networks across horizons, compared against Prophet in terms of MAE, RMSE, and NRMSE error metrics. In^12^, Mario Tovar et al. proposed a five-layer CNN-LSTM model for PV power predictions using real data for a location in Mexico. The results showed that the hybrid NN model has better predictions.

Maneesha P. et al.^7^ presented a forecast approach based on particle swarm optimization. Tests over four forecast resolutions and horizons revealed the proposed method reduced the mean absolute scaled error (MASE) by 3.81%. Grzebyk et al.^13^ proposed a single machine-learning model to forecast the power output of a large distributed solar fleet containing 1,102 PV systems. When evaluating the hourly forecast accuracy of the proposed XGBoost model at daily resolution, it achieved a mean absolute error (MAE) of 0.877 kW and a mean absolute percentage error (MAPE) of 23%. Ferlito et al.^14^ compared various machine learning methods with differing levels of complexity to determine if higher complexity models provide better forecasting performance. Additionally, they evaluated the considered techniques using both online and offline training methodologies to identify the most effective approach. Ibtihal A. A. et al.^15^ presented an ensemble stacked machine learning model for hourly forecasts of two photovoltaic systems differing in size and age. The study benchmarked three machine learning algorithms—random forest, gradient boosting, and multiple linear regressions—against a baseline linear regression model and the physical reference model for PV power prediction. Shadrack T. Asiedu et al.^16^ studied and compared the performance of single, ensembles, and hybrid machine learning models in predicting solar PV output power over four different time horizons (a day, a week, two weeks, and one month ahead). A summary of representative studies on PV power forecasting, including algorithms, dataset characteristics, and forecast horizons, is provided in Table 1.

**Table 1:** Summary of related work in PV power forecasting.

Reference	Method/algorithm	Dataset characteristics	Forecast horizon
Maneesha P. et al.^7^	Particle Swarm Optimization	PV forecasting data	4 different resolutions/horizons
Assouline et al.^8^	Support Vector Machine (SVM) with kernel technique	Urban Switzerland rooftop PV data	Not specified
Preda et al.^9^	SVM algorithm	Real sensor-generated data for hybrid renewable systems	Not specified
Muhammad W. et al.^10^	Extra Trees (ET) and Random Forest (RF)	Hourly PV power data	Hourly
Costa^11^	LSTM, CNN, and hybrid CNN-LSTM	Residential PV system data	Multiple horizons
Tovar et al.^12^	5-layer CNN-LSTM	Real PV data from Mexico	Not specified
Grzebyk et al.^13^	XGBoost	1,102 distributed PV systems	Hourly (daily resolution)
Ferlito et al.^14^	Various ML methods (complexity comparison)	PV forecasting data	Not specified
Ibtihal A.A. et al.^15^	Ensemble stacked ML (RF, GB, MLR)	Two PV systems (different sizes/ages)	Hourly
Asiedu et al.^16^	Single, ensemble, and hybrid ML models	Solar PV data	4 horizons (1 day, 1 week, 2 weeks, 1 month)
Present study	**Multi-label ML (LR**,** PR**,** ANN**,** DL**,** RF**,** GBT**,** DT**,** k-NN**,** SVM)**	**Real data from Cairo**,** Egypt - BAPV plant: 1-year**,** 5-minute intervals**,** meteorological + power data**	**Multiple (1 day**,** 1 week**,** 1 month)**

### Motivation

The increasing penetration of solar photovoltaic systems in power grids worldwide necessitates accurate forecasting capabilities to ensure grid stability and optimal energy management. Current grid operators face significant challenges in balancing supply and demand due to the intermittent nature of solar generation, which varies based on weather conditions, seasonal changes, and daily patterns. Effective forecasting models are crucial for enabling grid operators to make informed decisions regarding energy dispatch, storage utilization, and backup power activation.

### Research gap

Despite the considerable advances demonstrated in the literature, previous research has predominantly focused on single-label PV power forecasting approaches that predict only one output parameter. This limitation overlooks the potential benefits of simultaneously predicting multiple power system parameters, which could provide enhanced value for comprehensive system analysis. Incorporating a modeling approach that includes both PV and AC power predictions can provide additional value for applications such as grid integration analysis, inverter optimization, energy management systems, and financial planning algorithms for building-applied photovoltaic (BAPV) systems.

### Research objectives and contributions

This study addresses the identified research gap by developing and proposing multi-label forecasting models for predicting the PV array and AC power outputs of a BAPV plant. To analyse this multi-label forecasting framework, various machine learning algorithms (MLAs) spanning different model classes are implemented, including linear (LR) and polynomial regression (PR) models, artificial neural networks (ANNs), and deep learning (DL) models, random forests (RF), gradient-boosted trees (GBT), decision trees (DT), the K-nearest neighbour (k-NN) algorithm, and support vector machines (SVM). Meteorological variables such as solar irradiance (S_rad_-W/m^2^), ambient temperature (T_amb_-^o^C), and wind speed (W_s_-m/s) collected on-site using a weather station attached to the PV plant, along with cell temperature (T_cell_-^o^C) readings, are used as inputs. The measured PV array and AC power output values obtained from the BAPV plant data loggers are used as labels for the ML model. All algorithms are trained and tested on a high-frequency dataset covering one full year (from October 2022 to September 2023) of 5-minute interval measured plant performance data, ensuring the representation of different seasonal conditions. Afterward, the ML models are validated for short-, medium-, and long-term forecasts across multiple time horizons: 1 day (for sunny and cloudy conditions), 1 week, and 1 month ahead. Additionally, the models are also assessed for their ability to capture sudden variations in solar generation. For the prediction horizons in the year 2024 (one day, one week, and one month), each model was provided with actual measured weather inputs for the target periods, which were excluded from the training phase. The PV outputs for these periods were unknown to the models and were predicted solely from the unseen meteorological data. This setup allowed us to evaluate and compare the generalization performance of the different models under real measured weather conditions. All models are implemented and tested using the RapidMiner software environment.

### Paper organization

The paper is structured into four main sections: Sect. 2 presents the BAPV plant description; Sect. 3 outlines the methodology, including a description of the environmental and power plant parameters, the data pre-processing and feature selection techniques employed, and the evaluation metrics used to assess MLA performance. A detailed analysis and discussion of the results are presented in Sect. 4. Finally, Sect. 5 summarizes the key findings and conclusions of the study.

## BAPV power plant description

**Fig. 1:**
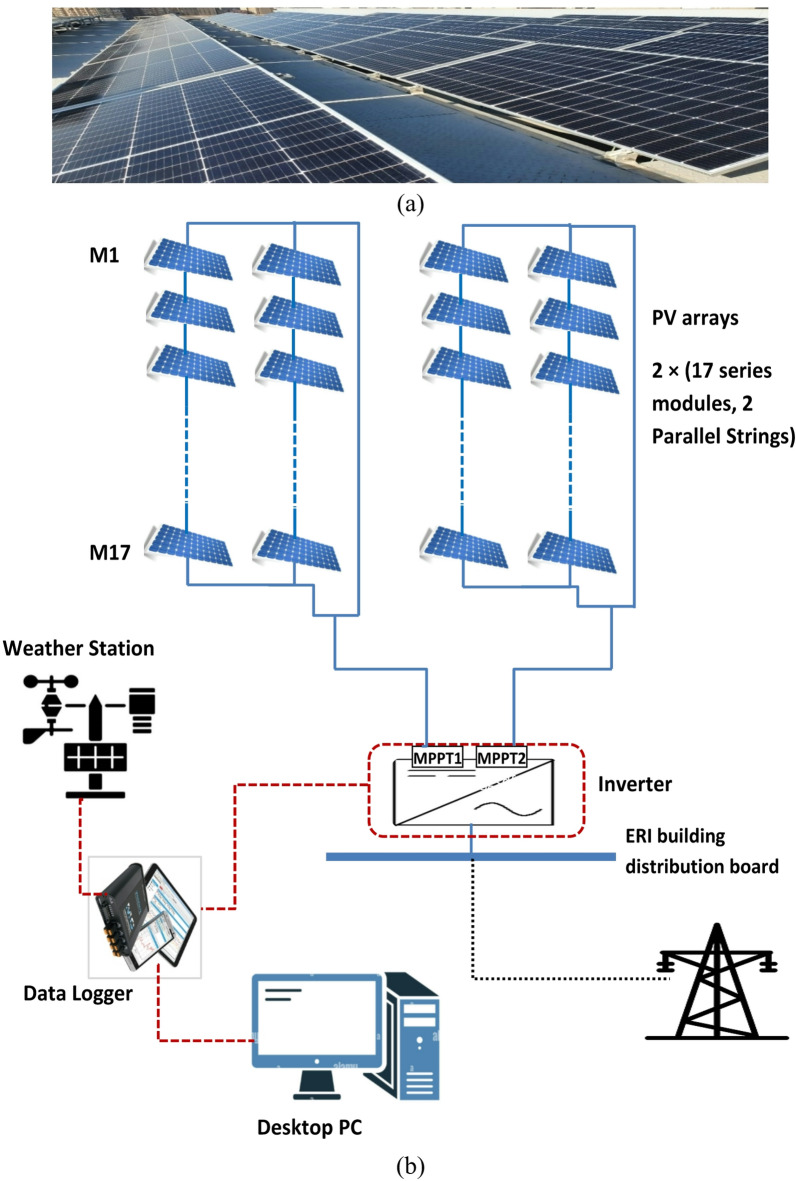
(**a**) PV array at ERI rooftop, (**b**) Schematic diagram of the PV plant.

The grid-connected BAPV system is located in New Nozha, Cairo, Egypt, at 30° 7’ 49.44’’ N latitude and 31° 22’ 48’’ E longitude, facing south (zero azimuth) with a fixed tilt angle of 26^o^ (Fig. 1a). The total capacity of the PV array system is 30.26 kW, arranged into two sub-arrays, each with a capacity of 15.13 kW. Each sub-array includes 34 (TallMax 445 W) panels arranged in two parallel strings of 17 modules in series (Fig. 1b). The mono-crystalline silicon PV module (TallMax-TSM-DE17M (II)) has a maximum power of 445 W and an efficiency of 20.4% under standard test conditions (STC) of 1000 W/m^2^ and 25 °C^17^.

These PV modules are connected to a 27.6 kW three-phase inverter (ABB TRIO-27.6-TL-OUTD) –Table 2 demonstrates all electrical characteristics of PV modules and the inverter. Meteorological data collected from an on-site weather station, alongside electrical parameter measurements, were systematically recorded using a Solar-Log Base 100 data logger. This data acquisition unit recorded measurements at regular intervals, functioning as the central monitoring device that interfaces the PV station and weather sensors. High temporal resolution data were gathered from the PV system and weather monitors, sampled at 5-minute intervals. The collected parameters included solar irradiance (S_rad_-W/m^2^), ambient temperature (T_amb_-^o^C), wind speed (W_s_-m/s), cell temperature (T_cell_-^o^C), as well as PV array output power (P_PV_) and AC power fed to the grid (P_AC_).

This dataset is transferred from the on-site monitoring equipment to a PC installed at the ERI building for long-term storage and analysis. With measurements logged every 5 min, intra-hour and short-term variations in solar generation and atmospheric conditions could be accurately tracked.

**Table 2:** Electrical specifications of the PV module and inverter^17^.

PV characteristics	
Module type	Mono-crystalline
Maximum power under STC (Wp)	445
Open-circuit voltage V_oc_ (V)	49.4
Short-circuit current Isc (A)	11.46
Efficiency (%)	20.4
Temperature coefficient of open-circuit voltage (%/^◦^C)	-0.26
Temperature coefficient of short circuit current (%/^◦^C)	-0.04
Temperature coefficient of power (%/^◦^C)	-0.36
Operating temperature	-40 ~ + 85 °C
Number of Cells	144 half-cut cells (6 × 24)
Inverter (DC/AC) characteristics	
Brand	ABB
Inverter model	TRIO-20.0/27.6-TL-OUTD
Number of independent MPPT channels	2
Maximum usable power for each MPPT channel (kW)	16
AC output voltage (V)	480
Nominal output frequency (Hz)	50/60
Ambient operating temperature range	-30 °C to + 60 °C
Total harmonic distortion	< 3%
Efficiency (Max/CEC) %	98.2/75.5

## Methodology

In this study, machine learning algorithms are employed to develop multi-label forecasts of the PV and AC power output for a BAPV plant. Different groups of machine learning algorithms and datasets are proposed to apply the forecasting model to ensure the accuracy and reliability of these models. The proposed MLAs consist of neural-based methods, including neural networks (NN), deep learning (DL), regression-based methods (linear regression (LR), tree-based methods including gradient-boosted trees (GBT), random forests (RF), and decision trees (DT), lazy-based methods (K-NN), and finally support vector machines (SVM).

RapidMiner Studio’s graphical user interface software is used in the modeling implementation. RapidMiner is a data analytics software platform created in 2001 by Ralf Klinkenberg, Ingo Mierswa, and Simon Fischer^18^. It offers a range of operators and repositories for processes like data preparation, transformation, modeling, and evaluation. Specific functionality includes tools for process control and utilities, repository access, importing/exporting, data manipulation, and model building. This research utilized Rapid Miner Studio version 10.1 Educational edition. Its comprehensive toolset is aimed to support the end-to-end data science workflow, covering aspects such as parameter tuning, model training, validation, and performance evaluation.

**Fig. 2:**
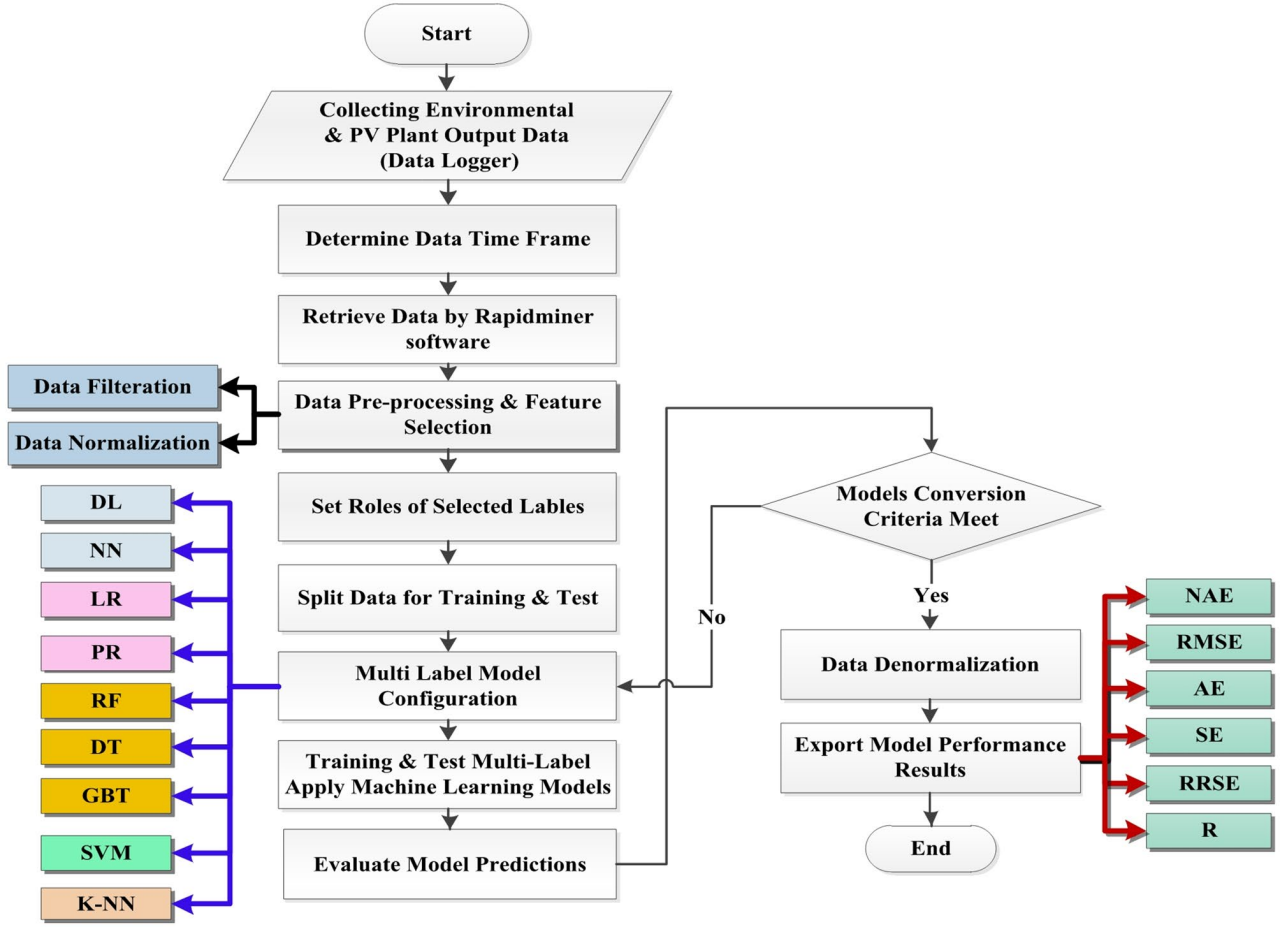
Flowchart of the proposed ML-based power forecasting methodology.

Figure 2 shows the flowchart implemented in this study, comprising input, training, and forecasting phases. The input phase involves data collection, providing environmental inputs of solar irradiance, ambient temperature, wind speed, and cell temperature measured on-site. Recorded PV and AC power outputs are the data labels to be predicted. Data pre-processing, discussed in Sect. 3.2, is then conducted. This important step refines the inputs before modeling to improve accuracy. During training, machine learning algorithms are developed using the pre-processed, historical input-output datasets. Models learn the relationships between inputs and targets to perform forecasts.

The forecasting phase allows for predicting PV and AC power production into the future. As new, real-time data is collected daily, models are continuously trained and used to issue multi-horizon power generation forecasts. The dataset used in the study is one year in length, ranging from October 2022 to September 2023, representing all seasons of the year, including winter, with high fluctuation in solar radiation and prediction complexity.

After the raw data were pre-processed, feature selection techniques such as feature importance ranking and Pearson correlation were then applied to identify the most predictive independent variables to include in the models. The pre-processed data was divided into training, validation, and test subsets using split sampling with a 70:30 ratio for training and testing, respectively. Machine learning algorithms were trained on the training set to iteratively learn patterns in the data and optimize model parameters through multiple iterations. Concurrently, hyper-parameter tuning using the validation set helped configure aspects of model structure not learned during training, such as the number of hidden layers in an NN and the kernel number in SVM. Once fully specified, the trained machine learning models were evaluated on their predictive performance using previously unseen observations from the held-out test set, to objectively measure how well the models generalize to new data. This workflow helps develop robust and optimized statistical learning models for reliable predictive accuracy assessments.

As part of the supervised learning process, the models developed using each MLA experienced further refinement and performance evaluation on independent validation data. The internal parameters of the algorithms were fine-tuned to optimize predictive accuracy. A variety of error and correlation metrics were then computed to assess and compare forecast quality across MLAs. These included absolute error (AE), root mean square error (RMSE), normalized absolute error (NAE), relative error (RE), relative root square error (RRSE), and correlation coefficient (R).

The workflow steps are summarized as follows:

- The PV plant data are collected through the weather station and data logger. This includes all meteorological data and measured outputs of the PV plant over one year with a 5-minute resolution.
- The data are prepared according to the required forecasting horizon (short, medium, or long-term).
- RapidMiner software is used to implement the MLAs. The data are retrieved for training and validation.
- Data filtering is performed to remove outlier values.
- Feature selection identified important input features and labels to predict.
- A data normalization technique is chosen based on the ML modeling approach.
- The pre-processed data was split into 70% for training and 30% for testing subsets.
- A multi-label machine learning models are applied to forecast AC power and PV power outputs.
- A validation model is then applied to a new dataset based on multiple time horizons to verify the model.
- The performance indicators of the model are evaluated and printed.

### Environmental parameters and data collection

The relationship between meteorological factors and photovoltaic (PV) power output is location-dependent, dictated by geographical and climatic conditions. Consequently, the degree of correlation between weather inputs and PV generation differs between sites. However, forecasting model accuracy hinges on the input-output correlation structure^19^, and a site-tailored approach is necessary. Different factors affect PV forecasting accuracy, making such predictions a sophisticated process. It depends on factors such as forecasting horizons, forecast model inputs, and performance estimation^20^.

Meteorological parameters play a pivotal role in PV power forecasting performance, as they directly influence generation levels. The most significant input is solar irradiance, followed by ambient temperature, as both are strongly correlated with PV output. The movement of clouds also determines sudden and abrupt changes in PV power production. This study selects all key meteorological factors that comprehensively represent site conditions as input features. Solar irradiance is the dominant parameter, given its direct energy relationship. Ambient temperature further impacts efficiency, especially at higher levels. Cell temperature and wind speed round out the input set. The performance of a prediction model is directly affected by the season of the year^4^; thus, seasonal effects necessitate balanced data representation during model training. As performance varies by weather patterns throughout the year, an equally distributed dataset covering all four seasons was used. This improves model generalization beyond any single season and avoids bias. Also, optimizing input selection is important to maximize accuracy while constraining computational overhead. In this case, all primary meteorological drivers are incorporated as inputs to provide a holistic view of influencing conditions impacting PV generation. Seasonal weighting also strengthens model robustness.

In this study, the meteorological data are measured from Oct 2022 to Sept 2023. This dataset comprises eight attributes, including date, time, radiation, ambient temperature, wind speed, cell temperature, PV power, and AC power. Data was recorded via the data logger at five-minute intervals (comprising more than 105 K data samples per parameter). The initial dataset is split to be trained and tested (70%-30%). The original dataset is measured every 5-minute record, hourly, and daily measurements. In this study, all meteorological parameters that affect PV production are selected as input features; this includes solar irradiance as a dominant parameter, ambient temperature as the second factor that has an impact at high temperatures, cell temperature, and finally wind speed. The PV power and AC power are selected as the targets for the multi-label machine learning model.

**Fig. 3:**
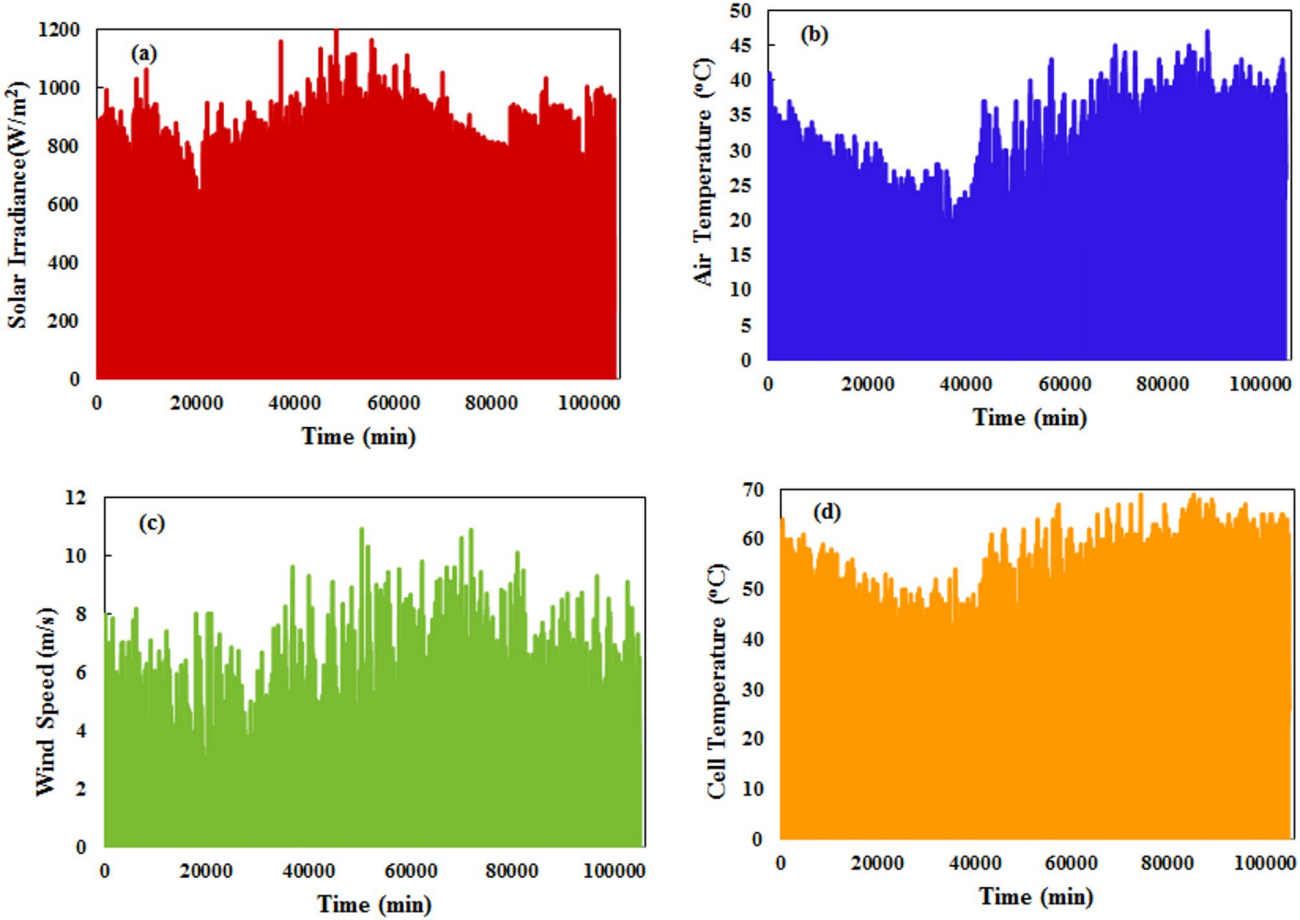
Full-year meteorological data recordings (**a**) S_rad_ (**b**) T_amb_, (**c**) W_s_, and (d) T_cell_.

Figure 3 demonstrates the variation in meteorological parameters and cell temperature over the selected year of the study. Solar irradiance changes from 0 to 1000 W/m^2^, with some minor readings up to 1200 W/m^2^ under clear weather conditions, especially in April and May. Ambient temperature varies from 10 to 44 °C, with minimum night temperatures near 10 °C and maximum daily temperatures approaching 44 °C. Cell temperature spans 10–68 °C. Wind speed fluctuates from 1 to 9 m/s, with repeating values at low levels, as Cairo has typical moderate to low wind speeds. Before modeling, this dataset goes through pre-processing to improve model accuracy. Pre-processing includes two main processes: filtration and data normalization. Outlier filtration removes odd readings while data normalization scales variables to the same measurement units, positioning data for optimal training. The appropriate data normalization methodology was selected according to the machine learning approach. As shown in Fig. 3, environmental patterns exhibit substantial seasonal and daily fluctuations influencing PV generation levels.

### Feature selection

Feature selection is an important step to identify the most influential input variables and discard irrelevant or redundant features. This helps develop a suitable feature subset for the model while improving performance and interpretability. In this study, the Pearson correlation coefficient^6^ was used to evaluate the relationship between each input predictor variable and the target (label) variable, due to the inherent characteristics of PV data. R is a widely applied metric of linear correlation between two quantitative variables, with a value between − 1 and 1. It indicates both the direction and magnitude of association - whether an increase in one variable tends to be accompanied by an increase or decrease in the other. Mathematically, the R between a feature x and target y is defined as the covariance of the two variables divided by the product of their standard deviations^21,22^:

1 $$\:R=\frac{\sum\:\left({x}_{i}-\bar{x}\right)\left({y}_{i}-\bar{y}\right)}{\sqrt{\sum\:{\left({x}_{i}-\bar{x}\right)}^{2}\sum\:{\left({y}_{i}-\bar{y}\right)}^{2}}}$$

Where, $\:{x}_{i}$ and $\:\bar{x}$ are the values and the mean of the x-variable in a dataset, respectively, $\:{y}_{i}$ and $\:\bar{y}$ are the values and the mean of the y-variable in a dataset, respectively. In our case, $\:{x}_{i}$ represents input features (S_rad_, T_amb_, W_s_, and T_cell_), while y represents output labels to be predicted (P_PV_, and P_AC_).

### Data pre-processing

Input solar power and meteorological data require pre-processing to enhance model accuracy and improve computational efficiency. Raw datasets may contain transient spikes and non-stationary components due to unpredictable weather. Issues like outliers, sparsity, and abnormal records are common and can interfere with modeling patterns. Pre-processing aims to refine datasets before ML model development. A series of filtering steps was applied to both photovoltaic power output and meteorological data. First, physically improbable values such as negative numbers, null readings due to sensor errors, or periods of missing sensor recordings were removed. Streamlining datasets aimed to reduce improper training problems and computational costs from irregularities, outliers, and irrelevant inputs. Providing full high-resolution datasets, including night-time null PV values, could negatively impact training and accuracy due to data sparsity. To address this, the night-time sampling frequency was reduced without a complete removal. Filters removed implausible outliers and days with extensive missing values.

In our study, a filtration strategy was applied to remove negative irradiance values, ensuring data consistency. Additionally, sparse night-time data, where PV power is null, was identified as a potential factor affecting model performance. To address this, the night-time dataset was reduced but not eliminated, as these data points represent the cyclic behavior of solar irradiation absence, which is essential for capturing realistic system dynamics. Furthermore, days with insufficient data records were filtered out to maintain dataset integrity and prevent inconsistencies during model training. By thoroughly cleaning and conditioning the datasets before model development, this strategy helped minimize potential training issues and computational burdens that incomplete or improper inputs may introduce. Pre-processing steps aimed to remove faulty inputs, address sparsity, isolate consistent patterns, and balance dataset properties for optimized machine learning. This facilitated proper learning of historical trends and enhanced forecasting performance.

The second step after applying filtration is data normalization. This study implements two data normalization techniques: range transformation and z-transformation. The selection criterion was based on the proposed machine learning algorithm. Range transformation normalized data to a fixed range between 0 and 1. In the range normalization technique, the input data are rescaled to fall within a smaller common range from their original wider range. Specifically, all variables are scaled between 0 and 1. This approach helps reduce regression errors by restricting values to a narrow interval while maintaining correlations between input parameters. It prevents variables with inherently high values from dominating those with smaller scales. Normalizing data in this way further allows machine learning algorithms to treat each feature equally during training, improving both training speed and convergence. Variables standardized to the same scale can then be directly compared in terms of their relative impact.

Mathematically, the min-max scalar transforms data according to the formula^23^:

2 $$\:\widehat{zs}=\frac{zi-{z}_{min}}{{z}_{max}-{z}_{min}}$$

Where,$\:\:\widehat{zs}$ is the scaled value, $\:zi$ is the measured value, $\:{z}_{max}$ and $\:{z}_{min}$ are the maximum and the minimum values of the dataset.

Range normalization rescales input data to a standardized interval; it transforms a wider range of data values into a restricted range between zero and one. In this study, the “min-max scalar” method is employed for range normalization. This normalization provides data rescaling to a constrained range that minimizes regression errors, preserves correlations within datasets, and improves precision. Moreover, range normalization addresses scale differences among input features that could otherwise dominate the modeling process. By standardizing to a common scale, variances in value magnitudes between parameters are mitigated. As a result, all inputs can be treated equally during training without some features overriding others. This optimized weighting boosts algorithm calculation speeds and convergence. The range normalization is used for all models tested in this study except support vector machines.

SVM instead used z-transformation, which standardizes variables by removing the mean and scaling them to unit variance. Compared to min-max normalization, the z-score transformation provides more accurate predictive results when using SVMs. The approach of standardizing by mean and variance yields superior model performance for SVMs versus other machine learning algorithms. This processing fits the SVM algorithm, which relies on calculating distances between samples. This normalization is a commonly used technique to standardize data values. It transforms values by subtracting the original mean and dividing by the standard deviation. The result is distributed data with a mean of 0 and a standard deviation of 1. This preserves the original shape of the distribution while making values dimensionless and comparable on the same scale^24^.

The z-score normalization formula applied to each value in the dataset is:

3 $$\:\widehat{zt}=(zi-\mu\:)/\sigma\:i$$

Where, $\:\mu\:$ is the mean of the data, and $\:\sigma\:i$ is the standard deviation of the data.

### Machine learning algorithms

#### Function-based algorithms

Regression analysis is a statistical method used for numerical prediction. It quantifies the relationship between a dependent or target variable (i.e., the label attribute) and multiple independent variables (regular attributes)^25,26^.

There are various regression techniques; two are used in this research:

- Linear regression (LR)

LR fits a straight line to capture the linear relationship between the dependent and independent variables. It models the observed data with a linear Eqs.^25,26^. The mathematical model that represents the I-V characteristics of PV as a function of (S_rad_, T_amb_, W_s_, and T_cell_) is given by:

4 $$\:{P}_{PV}=\eta\:\:A\:{\text{S}}_{\text{r}\text{a}\text{d}}\:\left(1-{\beta\:}_{T}\left({T}_{cell}-{T}_{ref}\right)\right)(1-\alpha\:{W}_{s})$$

The mathematical representation of PV predicted power using LR is as follows:

5 $$\:{P}_{PV}={\beta\:}_{0}+{\beta\:}_{1}{\text{S}}_{\text{r}\text{a}\text{d}}+{\beta\:}_{2}{\text{T}}_{\text{a}\text{m}\text{b}}+{\beta\:}_{3}{W}_{s}+{\beta\:}_{4}{T}_{cell}+ϵ$$

Where S_rad_, T_amb_, W_s_, and T_cell_ are input features. $\:{\beta\:}_{0}$ is the model intercept, $\:{\beta\:}_{1},\:{\beta\:}_{2},\:{\beta\:}_{3},\:{\beta\:}_{n}$ are the predictor coefficients. $\epsilon$ is the error term.

b. Polynomial regression (PR)

PR extends linear regression by incorporating nonlinear terms when the actual relationship is curved. It fits n^th^-order polynomial functions to the data (e.g., quadratic, cubic, etc.)^27,28^. The mathematical representation of PV predicted power using PR is as follows:

6 $$\:{P}_{PV}\:\:=\genfrac{}{}{0pt}{}{{{\beta\:}_{0}+\:\beta\:}_{1}{\text{S}}_{\text{r}\text{a}\text{d}}++{\beta\:}_{2}{S}_{rad}^{2}{+\beta\:}_{3}{\text{T}}_{\text{a}\text{m}\text{b}}+{\beta\:}_{4}{T}_{amb}^{2}+{\beta\:}_{5}{W}_{s}+{\beta\:}_{6}{W}_{s}^{2}{+\beta\:}_{7}{T}_{cell}{+\beta\:}_{8}{T}_{cell}^{2}}{\begin{array}{c}{+\beta\:}_{9\:}{{(\text{S}}_{\text{r}\text{a}\text{d}}\:.\:\text{T}}_{\text{a}\text{m}\text{b}}\left){+\beta\:}_{10\:}{{(\text{S}}_{\text{r}\text{a}\text{d}}\:.\:\text{T}}_{\text{c}\text{e}\text{l}\text{l}}\right){+\beta\:}_{11\:}{{(\text{S}}_{\text{r}\text{a}\text{d}}\:.\:\text{W}}_{\text{s}}\left){+\beta\:}_{12\:}{({\text{T}}_{\text{a}\text{m}\text{b}}\:.\:\text{T}}_{\text{c}\text{e}\text{l}\text{l}}\right)\\\:{+\beta\:}_{13\:}{({\text{T}}_{\text{a}\text{m}\text{b}}\:.\:\text{W}}_{\text{s}}\left){+\beta\:}_{14\:}{({\text{T}}_{\text{a}\text{m}\text{b}}\:.\:\text{T}}_{\text{c}\text{e}\text{l}\text{l}}\right){+\beta\:}_{13\:}{({\text{T}}_{\text{a}\text{m}\text{b}}\:.\:\text{W}}_{\text{s}}\left){+\beta\:}_{14\:}{({W}_{\text{s}}\:.\:\text{T}}_{\text{c}\text{e}\text{l}\text{l}}\right)+ϵ\end{array}}$$

#### Artificial neural network-based algorithms

Artificial neural networks (ANNs) are computational models inspired by biological neural circuits in the brain. An ANN contains interconnected units called neurons that process information via weighted links resembling synapses. Most commonly, ANNs are adaptive systems that can change their structure based on information flow during training. This enables them to model complex input-output relationships and discover hidden patterns in data. A feed-forward neural network is a basic ANN architecture where connections only transmit information in one direction, from input to output nodes, without cycles. Within this acyclic flow, data passes through one or more hidden layers of nodes that help extract higher-level features. During learning, feed-forward networks are presented with training examples, and weights are adjusted iteratively via back-propagation of error signals to minimize loss. This allows the network to gradually tune its synapse-like parameters until it can accurately map new inputs to predicted outputs^29^.

The back-propagation algorithm is a supervised learning technique for neural networks composed of two primary phases - propagation and weight updating. These phases operate through iterative training cycles. During propagation, input data is fed forward through the network to generate output predictions. The output values are then compared to true targets using an error function to quantify performance. Error signals derived from this analysis are then propagated backward through the network. This initiates the weight updating phase, where connection weights between nodes are adjusted in a direction that reduces the overall error. Repeated application of these two phases typically drives the network towards a stable state of minimal error, indicating it has learned the underlying relationships in the training data^26^.

A multilayer perceptron (MLP) is a type of artificial neural network well-suited for classification and regression problems. It employs a feed-forward architecture with one or more hidden layers of nodes situated between the input and output layers. All nodes are interconnected via bidirectional connections trained with back-propagation. Each node applies a nonlinear activation function, commonly sigmoid, to introduce nonlinearity. This allows MLPs to learn complex patterns across large input spaces^30,31^. In this work, two NN-based algorithms are implemented, which are:

- Neural network

The ML model architecture used in this study is a feed-forward multi-layer perceptron NN trained with a back-propagation algorithm. Within this framework, a standard sigmoid activation function is applied to the nodes. To suit the range expected by the sigmoid function, all input attributes were normalized to scale between − 1 and 1 using a normalization preprocessing step. Moreover, the output node uses either a sigmoid or linear activation function depending on the type of problem. Specifically, a sigmoid output activation was used since the task involves forecasting/classifying a variable, which represents a classification problem. On the other hand, a linear activation would be more appropriate for numeric regression tasks where the exact target value is to be predicted^32,33^. The mathematical representation of PV predicted power using NN is as follows:

7 $$\:{P}_{PV}={f}_{\text{out\:}}\left(\sum\:_{j=1}^{n}\:{\omega\:}_{j}^{\left(2\right)}\cdot\:{f}_{\text{h\:}}\left(\sum\:_{i=1}^{m}\:{\omega\:}_{ij}^{\left(1\right)}{x}_{i}+{b}_{j}^{\left(1\right)}\right)+{b}^{\left(2\right)}\right)$$

b. Deep learning

The deep Learning model^34–36^, in this work, is based on a multi-layer feed-forward artificial neural network that is trained with stochastic gradient descent with back-propagation. The deep Learning algorithm in Rapidminer uses H_2_O optimization^18^. The network contains a large number of hidden layers consisting of neurons with tanh, rectifier, and max-out activation functions. The mathematical representation of PV predicted power using DL is as follows:

8 $$\:{P}_{PV}={f}_{\text{out\:}}\left({{\upomega\:}}^{\left(L\right)}\cdot\:{f}^{(L-1)}\left({{\upomega\:}}^{(L-1)}\cdot\:\dots\:{f}^{\left(1\right)}\left({{\upomega\:}}^{\left(1\right)}\cdot\:\text{X}+{\text{b}}^{\left(1\right)}\right)+{\text{b}}^{(L-1)}\right)+{\text{b}}^{\left(L\right)}\right)$$

Where X is (S_rad_, T_amb_, W_s_, and T_cell_), $\:L$ Total number of layers in the deep neural network. $\:\varvec{\upomega\:}$ is the weight matrix for layer i. $\:{\text{b}}^{\left(i\right)}$is the bias vector for layer i. $\:{f}^{\left(i\right)}$ and $\:{f}_{\text{out\:}}$are activation functions for layer i and the output layer, respectively.

#### Trees-based algorithms

- Decision tree

A decision tree is a tree-based model that uses a hierarchical approach to classify or estimate target variables based on a set of input features or independent variables. The model contains a root node, internal decision nodes, and leaf nodes^18^. At each internal node, the data is split using a decision rule based on the value of a single predictive variable. The split separates the data into two or more homogeneous sets to be directed to the next child nodes. New child nodes are recursively generated from parent nodes until a stopping criterion is reached, such as a perfect split or a predefined depth limit. Predictions are determined depending on the majority class (for classification) or average value (for regression) within each terminal leaf node. For regression problems, the goal is to reduce the error of predicting a numerical target variable in the most optimal way. The hierarchical structure allows decision trees to model complex relationships between features and uncover interaction effects that may improve prediction performance. The mathematical representation of PV predicted power using DT is as follows:

9 $$\:{P}_{PV}=\sum\:_{i=1}^{N}\:{c}_{i}\cdot\:I\left(\text{X}\in\:{R}_{i}\right)$$

Where $\:{R}_{i}$ is a region in the feature space where the i^th^ rule applies.$\:\:{c}_{i}$ is a predicted constant value for the region $\:{R}_{i}$. $\:I\left(\text{X}\in\:{R}_{i}\right)$ is an indicator function, which is 1 if $\:\text{X}$ belongs to the region $\:{R}_{i}$ and 0.

b. Random forest

A random forest is an ensemble method that trains multiple decision trees on bootstrapped subsets of the original training data. The number of trees comprises a hyper-parameter called the “number of trees”. During training, each tree node represents a rule for splitting the data based on the optimal values of a random subset of predictors. This splitting criterion aims to reduce errors in target value estimation.

New nodes are recursively added in this splitting manner until meeting the stopping criteria. Only a fraction of available features specified by the “subset ratio” is considered at each potential split point to minimize correlation between trees. Once fully grown, the forest combines the predictions of its constituent trees via averaging (regression). This introduces diversity that mitigates over-fitting to any single training sample or feature subset, resulting in an accurate and robust predictive model^37–40^.

The mathematical representation of PV predicted power using RF is as follows:

10 $$\:{P}_{PV}=\frac{1}{T}\sum\:_{t=1}^{T}\:{P}_{PV}^{\left(t\right)}$$

11 $$\:{P}_{PV}^{\left(t\right)}=\sum\:_{i=1}^{N\left(t\right)}\:{{c}_{i}}^{\left(t\right)}\cdot\:I\left(\text{X}\in\:{{R}_{i}}^{\left(t\right)}\right)$$

Where $\:T$is the total number of DTs in the RF. $\:{P}_{PV}^{\left(t\right)}$ is the prediction from the t^th^ DT, which is computed using the rules of that tree.

$\:{{c}_{i}}^{\left(t\right)}$ is the constant value predicted by the t^th^ tree for the region $\:{{R}_{i}}^{\left(t\right)}$. $\:I\left(\text{X}\in\:{{R}_{i}}^{\left(t\right)}\right)$ is the Indicator function for whether $\:\text{X}$ belongs to the region $\:{{R}_{i}}^{\left(t\right)}$. $\:{{R}_{i}}^{\left(t\right)}$ is a region *i* of the feature space defined by the t^th^ tree.

c. Gradient-boosted trees

GBT is an ensemble learning technique that builds and combines weak prediction models sequentially to optimize predictive performance^41^. Like other boosted tree algorithms, it trains a series of decision tree models on adjusted versions of the training data. Specifically, trees are added one by one to minimize a differentiable loss function via gradient descent, providing a more structured and interpretable approach compared to other boosted tree techniques. This gradual, error-focused learning procedure improves the accuracy and controls the complexity of the trees to avoid over-fitting. While slower than a single model, the ensemble of tweaked decision trees combines to perform better than any single estimator^42–45^. The mathematical representation of PV predicted power using GBT is as follows:

12 $$\:{P}_{PV}={F}_{M}\left(\text{X}\right)=\sum\:_{m=1}^{M}\:\eta\:\cdot\:{T}_{m}\left(\text{X}\right)$$

Where $\:M$ is the total number of trees in the model.$\:\:{T}_{m}\left(\text{X}\right),\:$prediction from the $\:{M}^{th}$ decision tree.$\:\:\eta\:$ is the learning rate.

#### Lazy-based algorithms

Lazy learning algorithms, also known as instance-based algorithms, make predictions based on similarity to previously stored examples or instances, without explicit generalization.

- k-Nearest neighbor

The most common lazy learning model is the k-nearest neighbor (k-NN) algorithm. The k-NN algorithm makes predictions based on the closest training examples in a feature space^46^. During training, k-NN simply stores the feature vectors and corresponding target values and does not perform any explicit generalization. To classify a new example, k-NN finds the k closest examples already stored (based on a distance measure like Euclidean) and predicts the most common class among those neighbors. For regression, it averages the target variable values of the nearest neighbors to forecast a continuous value. Nearer neighbors may be weighted more heavily to influence the prediction. Feature values are often normalized before distance calculations to avoid biases from variations in scale. In this paper, common preprocessing min-max scaling is applied. An advantage of k-NN is that it is simple to implement and understand. A disadvantage is that it requires lots of memory to store all training examples and has a high computational cost for classification^16,47–50^. The mathematical representation of PV predicted power using K-NN is as follows:

13 $$\:{P}_{PV}=\frac{1}{k}\sum\:_{i=1}^{k}\:{P}_{PV}^{\left(i\right)}$$

For each training data, compute the distance $\:d\left(\text{X},{\text{X}}_{i}\right)$ between the features and the training point. A common distance metric is Euclidean distance, which is given by:

14 $$\:d\left(\text{X},{\text{X}}_{\text{i}}\right)=\sqrt{{\left({\text{S}}_{\text{r}\text{a}\text{d}}-{\text{S}}_{\text{r}\text{a}\text{d},\text{i}}\right)}^{2}+{\left({\text{T}}_{\text{a}\text{m}\text{b}}-{\text{T}}_{\text{a}\text{m}\text{b},\text{i}}\right)}^{2}+{\left({\text{T}}_{\text{c}\text{e}\text{l}\text{l}}-{\text{T}}_{\text{c}\text{e}\text{l}\text{l},\text{i}}\right)}^{2}+{\left({\text{W}}_{\text{s}}-{\text{W}}_{\text{s},\text{i}}\right)}^{2}}$$

#### Support vector machine-based algorithms

The model in this work uses the mySVM software package developed by Stefan Ruping for support vector machine (SVM) modeling. Specifically, it utilizes mySVM’s Java-based implementation, which restricts SVM modeling to a linear kernel function^18^. However, this linear kernel approach results in a parsimonious SVM model that only contains the linear coefficient terms. This more compact representation allows for faster deployment and application of the trained model compared to other SVM kernel types that have greater complexity. By leveraging mySVM’s efficient linear SVM formulation, the proposed methodology aims to balance predictive accuracy with reduced computational overhead for practical solar power forecasting applications. The mathematical representation of PV predicted power using SVM is as follows:

15 $$\:{P}_{PV}={{\upomega\:}}^{T}\text{X}+b$$

Where $\:\text{X}$ is the feature vector. $\:b$ is a bias term that shifts the decision boundary. $\:\varvec{\upomega\:}$ is a weight vector orthogonal to the decision hyperplane.

### Performance indices

In order to evaluate the performance of each machine learning algorithm (MLA) used in this work and assess their predictive accuracy, various statistical metrics are employed. These metrics include the absolute error (AE), root mean square error (RMSE), normalized absolute error (NAE), relative root square error (RRSE), and correlation coefficient (R). The Absolute Error is the default function of the RapidMiner software^51^.

16 $$\:AE=\:{y}_{p}-{y}_{i}$$

17 $$\:RMSE=\sqrt{\frac{1}{n}\:\sum\:_{t=1}^{n}{\left(\frac{\left({y}_{p}-{y}_{i}\right)}{{y}_{i}}\right)}^{2}}$$

18 $$\:RE=\frac{\left({y}_{p}-{y}_{i}\right)}{{y}_{i}}\:$$

19 $$\:NAE=\frac{1}{n}\:\sum\:_{t=1}^{n}\left|\frac{\left({y}_{p}-{y}_{i}\right)}{{y}_{i}}\right|$$

20 $$\:RRSE=\sqrt{\frac{\sum\:_{i=1}^{n}\:{\left({y}_{p}-{y}_{i}\right)}^{2}}{\sum\:_{i=1}^{n}\:({y}_{i}-\stackrel{-}{y}{)}^{2}}}$$

Where $\:{y}_{i}$ is the measured power value, $\:{y}_{p}$ is the predicted power value.

## Results and discussion

### Models train and test

In this study, we used weather data and cell temperature to train various MLAs for predicting PV and AC power. We utilized a one-year dataset for training and testing, employing RapidMiner software. Our main goal was to minimize the absolute error as the default function. After data pre-processing and feature selection, 70% of the dataset was used for training and validation, while the remaining 30% was kept for testing. The proposed MLAs include a neural-based method (NN, DL), regression-based methods (LR, PR), trees-based methods (GBT, RF, DT), and the k-NN) lazy-based method, and SVM.

The dataset used for training, testing, and validation consists of high-resolution measurements recorded at 5-minute intervals over a full year from 1st October 2022 to 30th September 2023 (30.26 kW PAPV plant), comprising 105,000 data samples. This ensures the model captures various seasonal and environmental variations. The training and testing phases utilize the entire year’s data to establish robust learning patterns across different weather conditions. For forecasting, validation is performed using only input weather variables—solar radiation, ambient temperature, cell temperature, and wind speed—without direct knowledge of future power output. The model then predicts PV power for different horizons, including short-term (one-day), medium-term (one-week), and long-term (one-month) forecasts. This approach allows the model to generalize effectively across time horizons while maintaining accuracy by leveraging historical relationships between meteorological variables and PV output.

- Short-term horizon: A 24-hour input period, including one sunny day and one cloudy day in 2024 (dataset: 288 samples/parameter).
- Medium-term horizon: A seven-day input period in 2024 (dataset: 2016 samples/parameter).
- Long-term horizon: A one-month (30-day) input period in 2024 (dataset: 7823 samples/parameter).

The data is normalized to a range of [0, 1]. Normalizing the data has the advantage of eliminating the impact of data units on the model, speeding up convergence, and reducing training time during the learning process. The performance test set is used to assess the accuracy of the model’s forecasts. The PC specifications used to execute the software: Intel^®^ core™ i7-4702MQ – 2.2 GHz, RAM 8 GB.

**Fig. 4:**
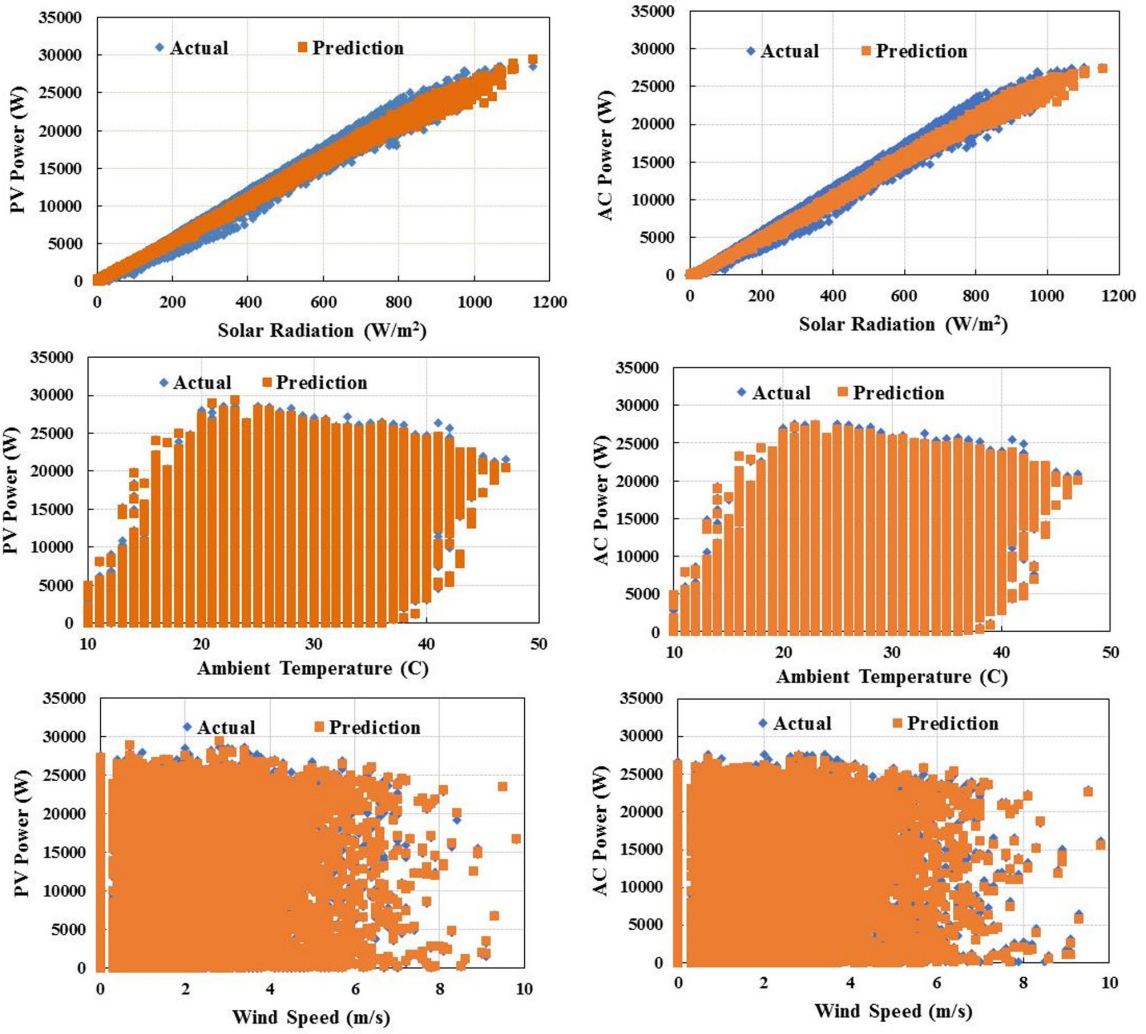
PV and AC power variation with environmental conditions based on RF algorithm prediction.

**Fig. 5:**
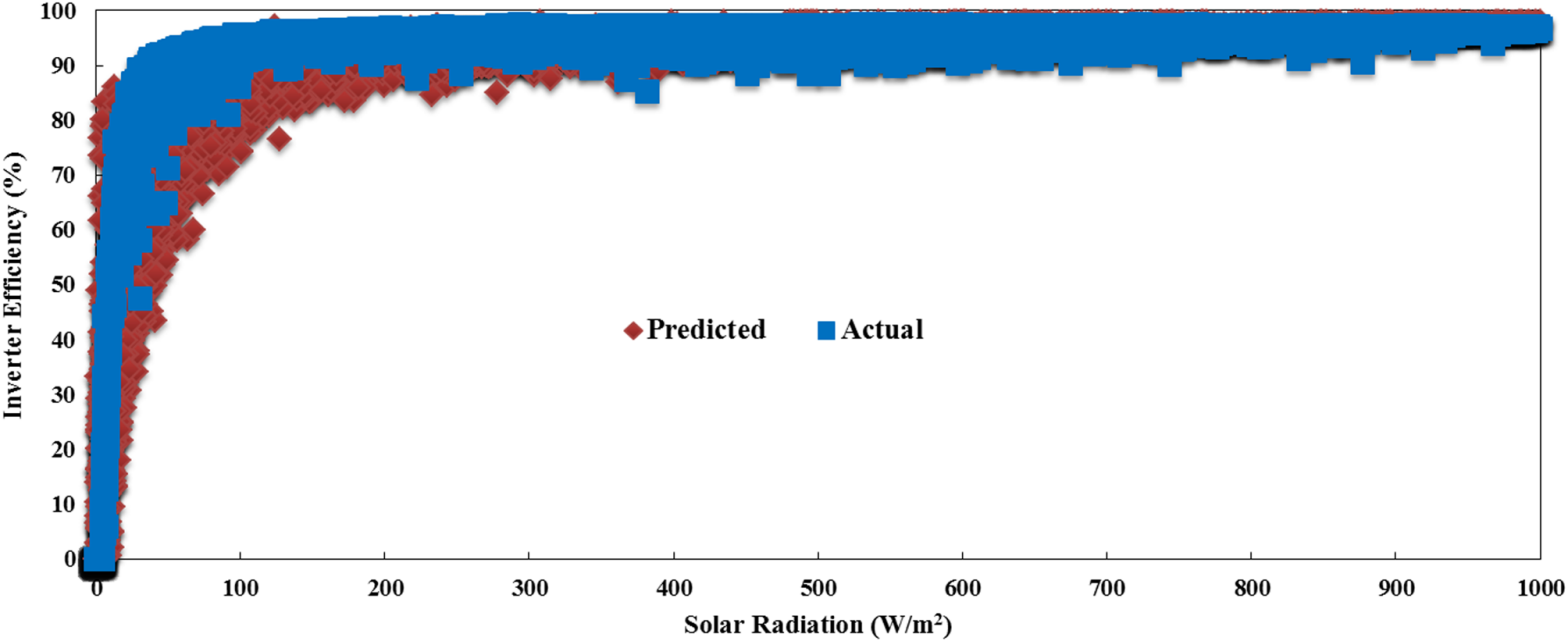
Actual and predicted inverter efficiency based on the RF algorithm.

Table 3 shows the evaluation indices of the tested MLAs. Based on the performance metrics presented in the table, our analysis reveals that the top-performing MLAs consist of RF, DL, and DT, which consistently achieve superior accuracy with RMSE values between 0.022 and 0.023, absolute errors of 0.015 and 0.016, and excellent correlation coefficients of 99.7%. These algorithms demonstrate remarkable precision in PV power forecasting, maintaining relative errors between 6.04% and 12.48%, significantly lower than their counterparts. LR, NN, and K-NN still deliver strong results with slightly higher error metrics (RMSE: 0.024–0.025, AE: 0.017) while maintaining excellent correlation coefficients between 99.6% and 99.7%. However, their relative errors show greater variation, ranging from 12.26% to 33.65%. While SVM, GBT, and PR demonstrate substantially higher and relative errors, PR is particularly underperforming across all metrics. DT offers the optimal balance with the fastest execution time (1 s) while achieving near-best performance across all metrics, including the lowest relative error (6.04%) among all algorithms. These results have critical practical implications for grid operations across different timeframes. DT offers the ideal balance of speed and accuracy for real-time grid operations, with its 1-second execution time and RE of 6.04% providing grid operators with highly reliable short-term forecasts that can be updated frequently. Figure 4 compares the yearly predicted PV and AC power output to the actual output, including environmental variables. This figure is for the RF algorithm, which has the best performance. As shown, the actual and predicted values follow a similar trend throughout the different seasons and times of the year. Figure 5 highlights the benefits of predicting both PV and AC power output together. It shows the actual and predicted inverter efficiency. Both values follow about the same trend, with only minor differences at low radiation levels. However, at high radiation levels, the actual and predicted inverter efficiencies are almost identical. In that case, the actual and predicted average efficiencies of the inverter are 0.96688 and 0.9638, respectively.

**Table 3:** The performance indices of the MLAs.

MLA	Execution time (sec)	RMSE	AE	RE (%)	RRSE	NAE	*R* (%)
LR	3	0.025	0.017	12.26	0.088	0.069	99.6
PR	43	0.238	0.119	797.23	0.836	0.471	75.1
GBT	6	0.106	0.094	665.77	0.374	0.371	99.7
RF	20	0.022	0.015	7.32	0.076	0.06	99.7
DT	1	0.023	0.016	6.04	0.081	0.063	99.7
NN	12	0.024	0.017	33.65	0.083	0.069	99.7
DL	18	0.022	0.015	12.48	0.076	0.061	99.7
K-NN	39	0.024	0.017	17.31	0.083	0.066	99.7
SVM	149	0.091	0.063	39.35	0.091	0.071	99.6

### Multiple forecasting horizons validation

This section evaluates the performance of different MLAs (NN, DL, LR, GBT, RF, DT, NN, and SVM) for different forecasting horizons under varying weather conditions.

**Fig. 6:**
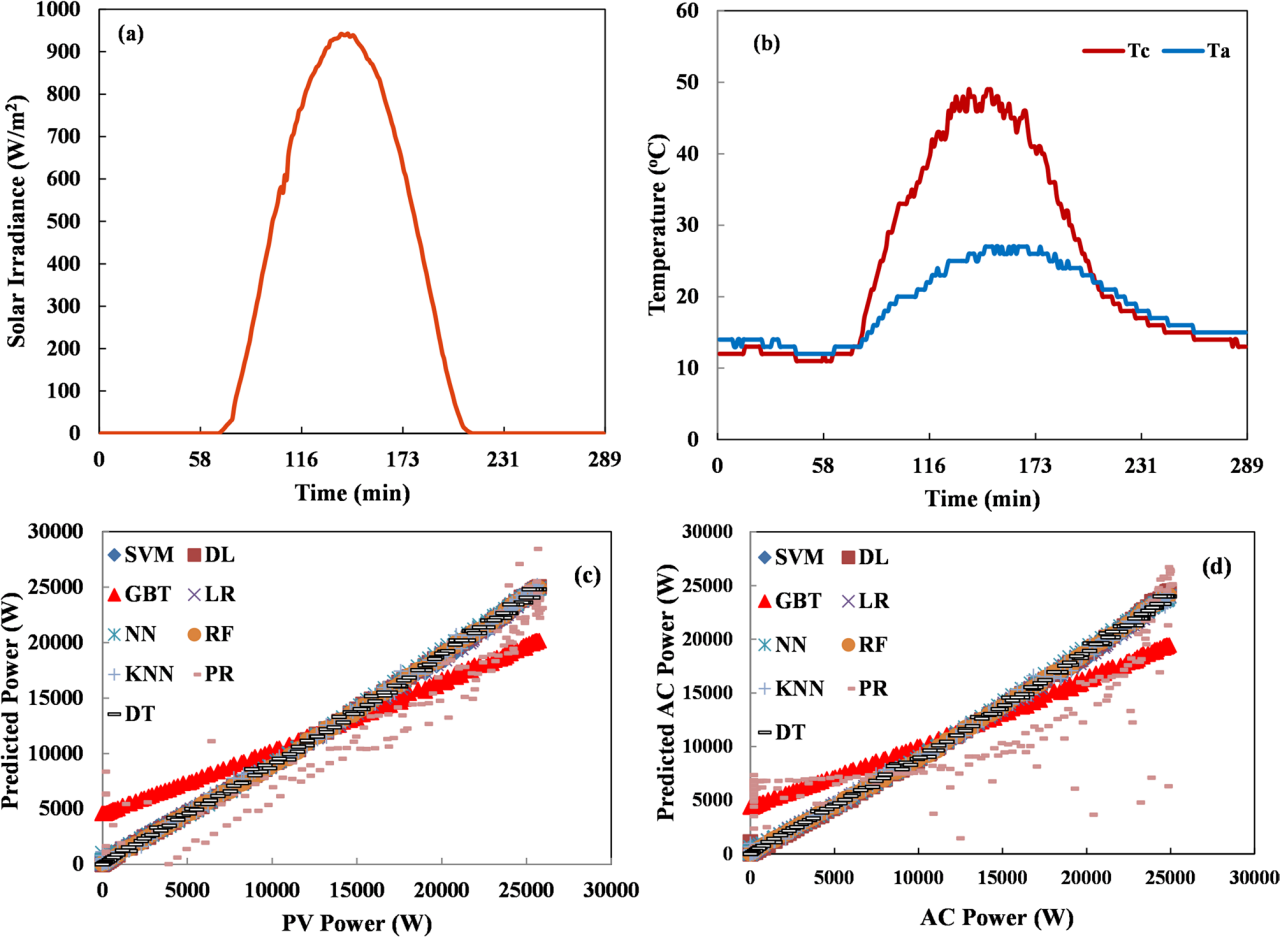
Short-term power forecasting of a sunny day (**a**) solar radiation, (**b**) Temperature (cell and ambient), (**c**) PV power variation, and (**d**) AC power variation.

For short-term forecasting, validation data from a sunny day and a cloudy day were used in this model. Figure 6 compares the measured and predicted power outputs from all models for a sunny day, representing the short-term forecasting horizon. The prediction data used in this test contained 288 values at 5-minute intervals. All models achieved a good level of accuracy in predicting power output, whereas the results seemed similar across all models, with slightly higher accuracy for the NN and RF models. The RF model provided the best results. Table 4 shows the average evaluation metrics of the MLAs for the sunny day validation data. The table shows that the sunny day performance data demonstrates the superiority of RF, DL, DT, and NN algorithms. Under sunny conditions, these algorithms achieve RMSE values between 0.023 and 0.026 and absolute errors between 0.015 and 0.018, confirming their reliability across different weather patterns. Notably, all four algorithms achieve perfect correlation coefficients (*R* = 100%) except for DL (99.9%), indicating exceptional pattern-matching capabilities during clear sky conditions when solar irradiance follows more predictable daytime patterns. A key finding emerges when examining relative error (RE) metrics for sunny day forecasting. DT demonstrates remarkable RE of just 8.91%, substantially outperforming other MLAs. Even more impressive is K-NN, which achieves the lowest RE at 5.39%. This suggests that K-NN’s instance-based learning approach may be particularly effective at capturing the characteristic patterns of sunny day PV generation. RF maintains strong performance with 14.57% RE. NN shows a somewhat higher relative error at 50.29% despite its low RMSE and perfect correlation coefficient. These results have vital practical implications for grid operators and energy market participants. During sunny days when PV production is at its peak, selecting a forecasting algorithm significantly impacts prediction accuracy. For real-time operations requiring frequent forecast updates, DT offers an optimal combination of speed (1 s) and accuracy (RE: 8.91%). The perfect correlation coefficients achieved by several algorithms (LR, GBT, RF, DT, NN) during sunny days indicate that they all capture the fundamental diurnal pattern of solar production under clear conditions.

**Table 4:** The evaluation indices of the mlas models for a sunny day.

MLA	Execution time (sec)	RMSE	AE	RE (%)	RRSE	NAE	*R* (%)
LR	1	0.026	0.017	18.03	0.076	0.057	100
PR	46	0.188	0.159	720.46	0.549	0.519	84.5
GBT	2	0.147	0.139	1296.76	0.429	0.455	100
RF	22	0.023	0.015	14.57	0.069	0.050	100
DT	1	0.024	0.015	8.91	0.071	0.049	100
NN	5	0.023	0.018	50.29	0.066	0.059	100
DL	17	0.026	0.018	42.62	0.07	0.051	99.9
K-NN	45	0.023	0.016	5.39	0.075	0.059	99.7
SVM	178	0.104	0.081	21.9	0.086	0.075	99.9

**Fig. 7:**
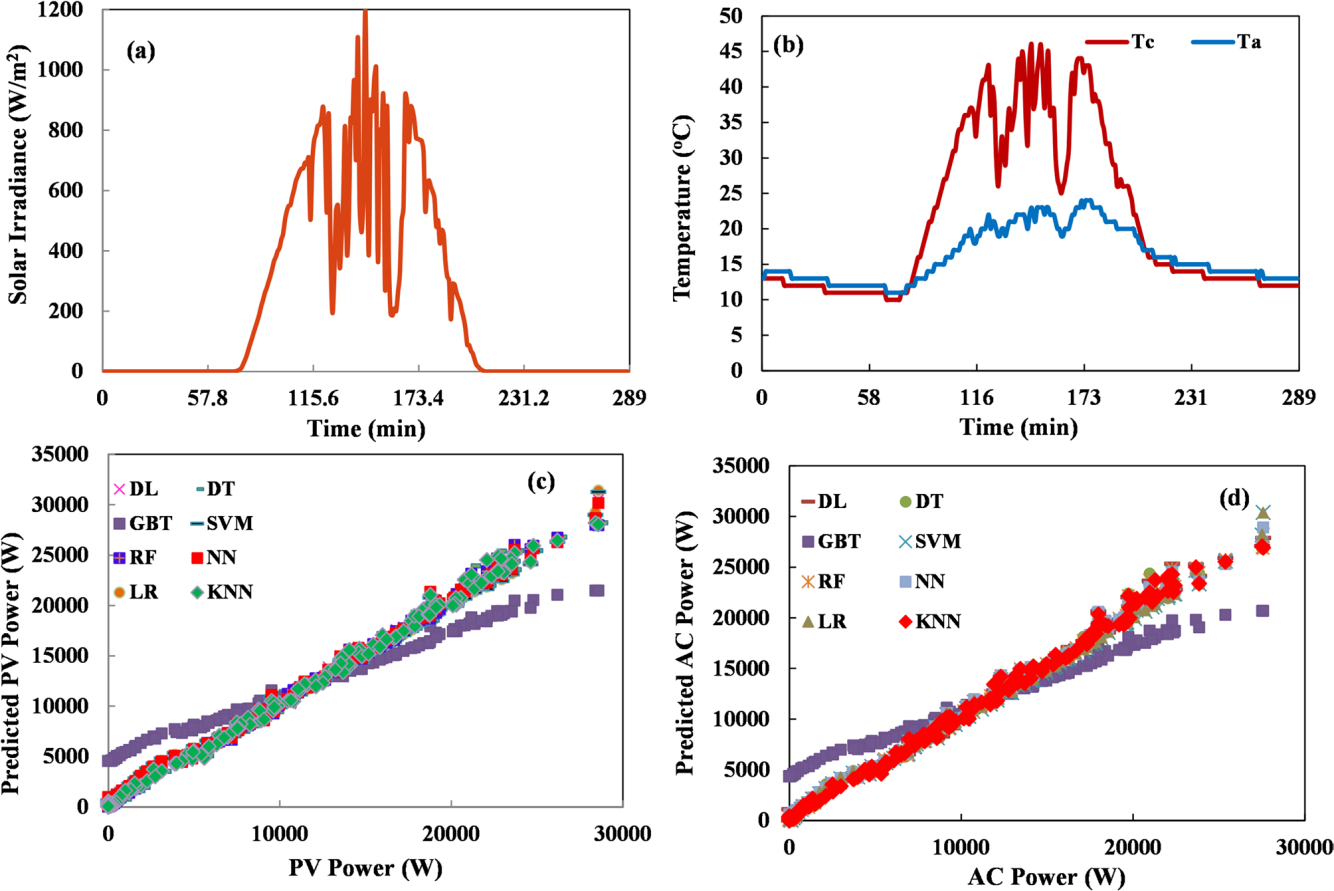
Short-term power forecasting of a cloudy day (**a**) solar radiation, (**b**) Temperature (cell and ambient), (**c**) PV power variation, and (**d**) AC power variation.

Figure 7 shows that the power output values forecasted by the MLAs using cloudy day (288 samples at 5-minute intervals) validation data are very close to each other and have good agreement with the measured values. The error metrics RMSE, AE, and RE are also small. The performance of the DL, DT, and RF models was superior to the other models, especially under highly variable weather conditions like a cloudy day. Table 5 presents the evaluation metrics of the different MLA models for the cloudy day validation data. This table reveals a difference in MLAs’ effectiveness compared to sunny conditions. LR and DT emerge as influential performers during cloudy days, achieving the lowest RMSE (0.016 and 0.018) and AE (0.009) values while maintaining speedy execution times of just 1 s each. The relative error (RE) patterns during cloudy conditions show that DT performs well with 11.27% RE, followed closely by RF at 17.08%. The substantial improvement in SVM’s relative error suggests it may be particularly adept at handling the complex, non-linear relationships characterizing cloudy day PV generation. Most algorithms maintain high correlation coefficients (*R* ≥ 99.9%) during cloudy conditions, with only PR showing a significantly lower correlation (84.5%). This indicates that most algorithms successfully capture the temporal patterns of PV generation even under variable cloud conditions. However, the absolute and relative error metrics reveal important differences in prediction magnitude accuracy. GBT and PR continue demonstrating poor performance with exceptionally high relative errors (1332.01% and 1142%, respectively), confirming their unsuitability for PV forecasting across weather conditions. The cloudy day analysis suggests that simpler, faster algorithms like LR and DT are preferable for forecasting during variable cloud conditions, offering both superior accuracy and computational efficiency.

**Table 5:** The evaluation indices of the mlas models for a cloudy day.

MLA	Execution time (sec)	RMSE	AE	RE (%)	RRSE	NAE	*R* (%)
LR	1	0.016	0.009	35.62	0.057	0.036	99.9
PR	37	0.188	0.168	1142	0.671	0.698	84.5
GBT	1	0.134	0.124	1332.01	0.478	0.513	99.9
RF	14	0.018	0.010	17.08	0.064	0.040	99.9
DT	1	0.018	0.009	11.27	0.065	0.036	99.9
NN	4	0.019	0.013	93.85	0.066	0.056	99.9
DL	14	0.018	0.010	43.87	0.065	0.040	99.9
K-NN	26	0.019	0.011	5.39	0.067	0.048	99.9
SVM	147	0.061	0.046	8.84	0.062	0.055	99.98

**Fig. 8:**
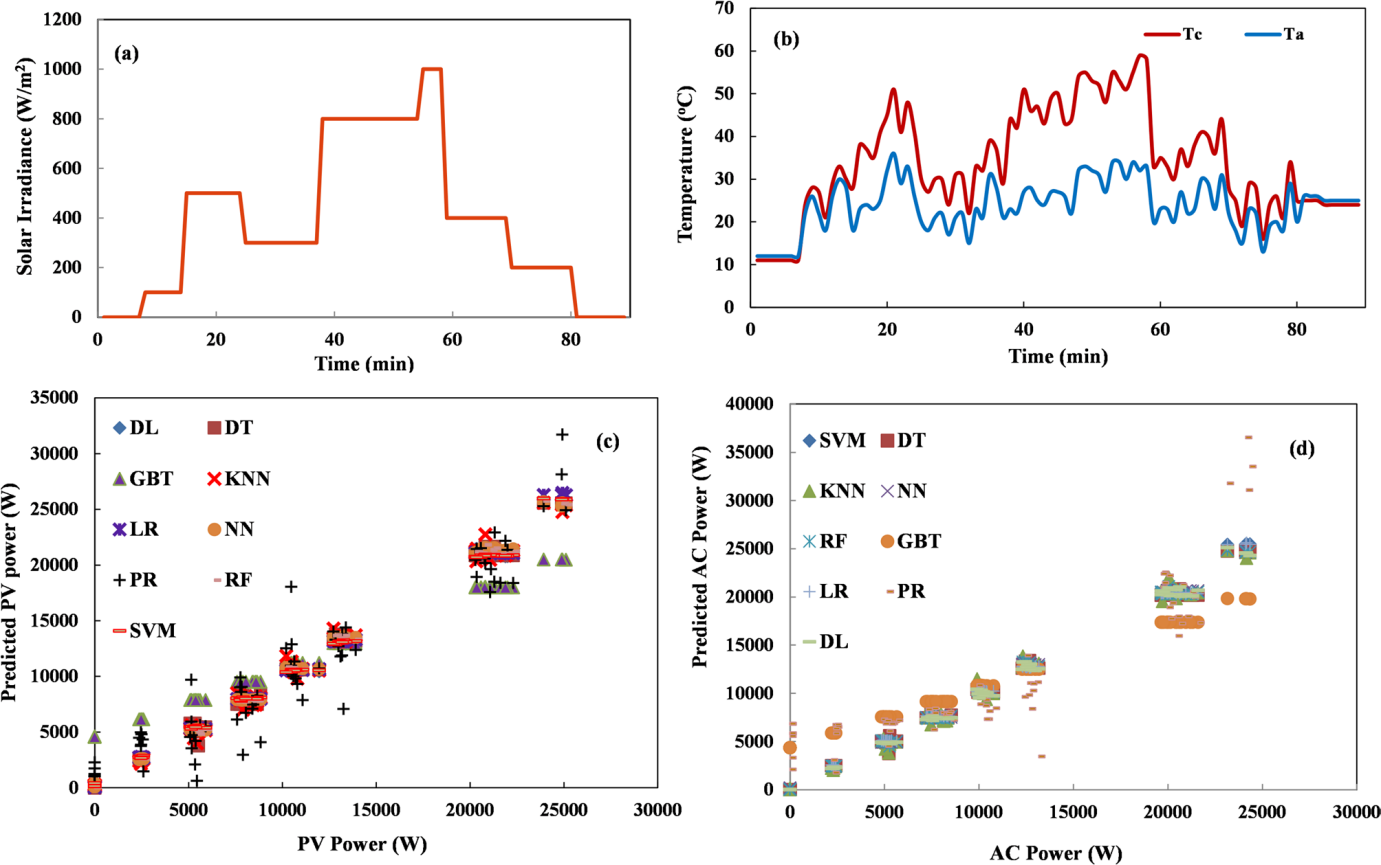
Short-term power forecasting with sudden variation (**a**) solar radiation, (**b**) Temperature (cell and ambient), (**c**) PV power variation, and (d) AC power variation.

Having evaluated short-term forecasting performance for full sunny and cloudy days, further analysis was done to assess the models’ accuracy during sudden changes in conditions. In this test, the validation data contained 90 records where solar radiation suddenly varied between 200 and 1000 W/m^2^, incrementing/decrementing by 200 W/m^2^ each time (Fig. 8). This tested the models’ ability to predict abrupt shifts in radiation levels. The results showed very accurate forecasting by all the MLAs and programs in this step-change scenario. NN and RF models demonstrated the best prediction accuracy. As Table 6 shows, the evaluation metrics for sudden variations in solar radiation represent the most critical test case for PV forecasting algorithms, as these rapid transitions from low to high irradiance (or vice versa) present significant challenges for grid stability and economic dispatch. LR demonstrates strong performance during sudden variations, achieving an RMSE of 0.018 and an AE of 0.012, with the lowest relative error among all algorithms at just 3.79%. This is particularly noteworthy given LR’s computational efficiency (1-second execution time) and relative simplicity compared to more complex algorithms. NN also gives an impressive RE of 3.61%, suggesting its ability to capture complex non-linear patterns becomes particularly valuable during rapid irradiance transitions. RF maintains its strong overall performance with low RMSE (0.017) and AE (0.012) values, though its RE (3.98%) is slightly higher than LR and NN. DT and DL follow closely with similar error metrics and excellent correlation coefficients, demonstrating their reliability across varied conditions. For grid operators concerned with ramp events and sudden production changes, these results suggest that simpler algorithms like LR and NN offer excellent performance for detecting and predicting rapid transitions in PV output while maintaining high computational efficiency.

**Table 6:** The evaluation indices of the mlas models for sudden variation.

MLA	Execution time (sec)	RMSE	AE	RE (%)	RRSE	NAE	*R* (%)
LR	1	0.018	0.012	3.79	0.068	0.053	99.98
PR	45	0.134	0.094	28.8	0.496	0.418	88
GBT	2	0.102	0.087	29.97	0.378	0.386	99.9
RF	19	0.017	0.012	3.98	0.064	0.055	99.8
DT	1	0.019	0.013	4.63	0.069	0.058	99.8
NN	5	0.017	0.012	3.61	0.061	0.055	99.8
DL	16	0.019	0.014	4.27	0.07	0.06	99.8
K-NN	29	0.023	0.016	5.39	0.084	0.073	99.7
SVM	73	0.063	0.044	11.4	0.067	0.056	99.8

**Fig. 9:**
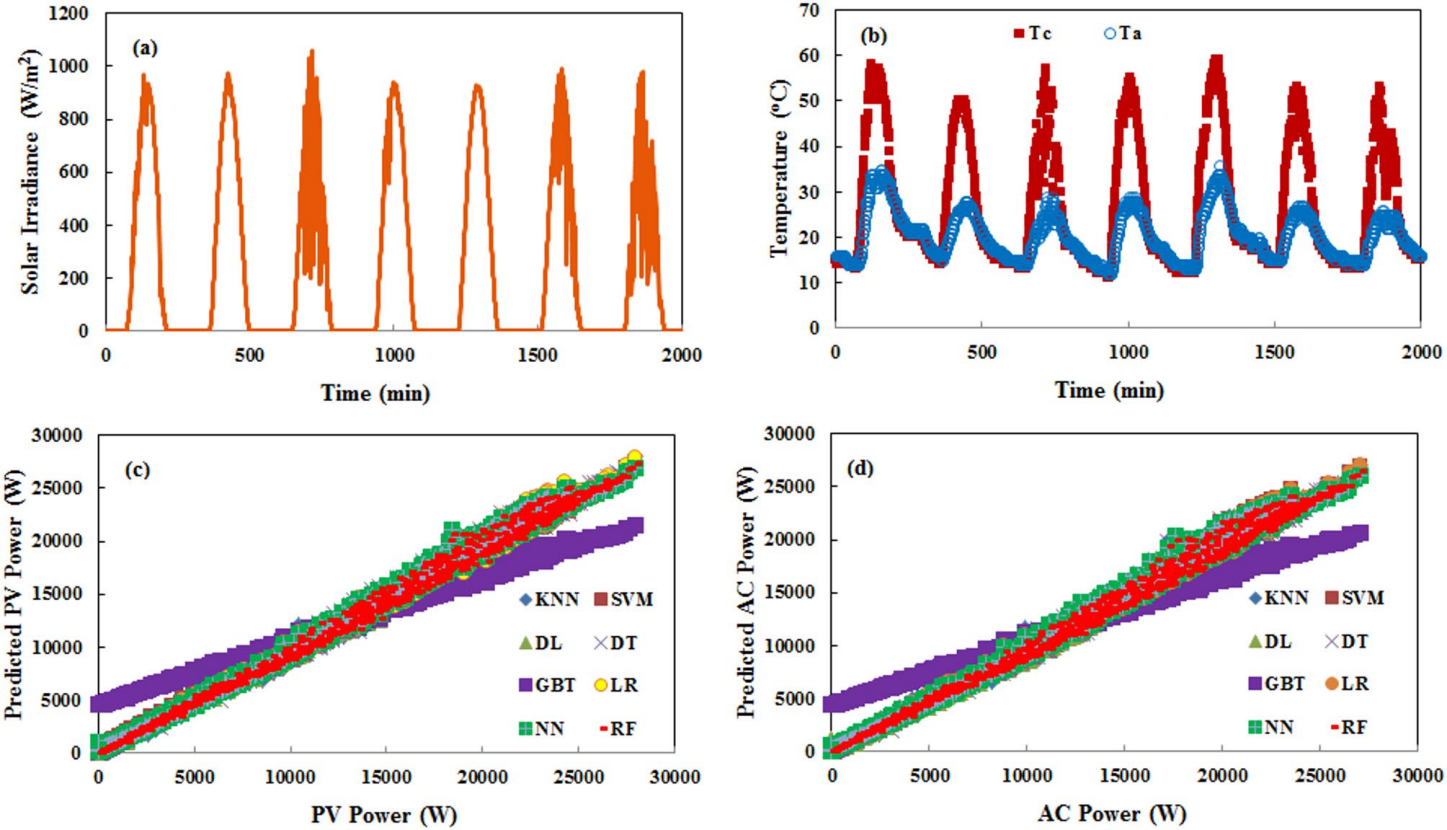
Medium-term power forecasting for one week (**a**) solar radiation, (**b**) Temperature (cell and ambient), (**c**) PV power variation, and (**d**) AC power variation.

For validating model performance at the medium-term forecasting horizon, data spanning a full week was utilized, consisting of 2016 samples. Figure 9 depicts a comparison between measured PV plant output and the predicted values generated by all machine learning models under evaluation. Various error and correlation metrics were calculated to summarize and compare the forecasting accuracy attained by each machine learning algorithm, with results presented in Table 7. The evaluation metrics for one-week time horizon forecasting provide valuable insights into algorithm performance for medium-term PV prediction, which is crucial for weekly energy market participation and operational planning. This analysis reveals distinct patterns in prediction accuracy and computational efficiency for this important forecasting timeframe. DT is particularly impressive for one-week forecasting, achieving the lowest relative error (7.79%) while maintaining the fastest execution time (1 s) along with LR. LR and RF also demonstrate excellent performance with low RMSE values (0.018) and absolute errors (0.011), though their relative errors (19.48% and 14.95%, respectively) are somewhat higher than DT. NN, DL, and K-NN have similar RMSE values between 0.017 and 0.020 and identical absolute errors of 0.012. However, their relative errors vary significantly—DL achieves 17.17% RE, while NN and K-NN show higher values around 62%. This disparity between absolute and relative error metrics suggests these algorithms may struggle with consistent magnitude prediction across different production levels in the week-long horizon, despite capturing temporal patterns accurately. SVM presents an interesting case for weekly forecasting, achieving a competitive relative error (13.58%) with reasonable computational efficiency (5 s), representing its best performance across all analyzed scenarios. This suggests SVM may be particularly well-suited for capturing the complex patterns spanning multiple days within a week. GBT and PR continue their pattern of poor performance, with PR showing an extremely high relative error (991.58%) and GBT exhibiting an RE value of 153,521%, indicating complete unsuitability for week-long forecasting. This analysis suggests DT for practical applications, which offers the optimal combination of accuracy and computational efficiency. DT provides reliable predictions with minimal computational overhead, while RF offers slightly improved absolute accuracy at a higher computational cost for applications where execution time is less constrained.

**Table 7:** The evaluation indices of the mlas models for one-week time horizon.

MLA	Execution time (sec)	RMSE	AE	RE (%)	RRSE	NAE	*R* (%)
LR	1	0.018	0.011	19.48	0.058	0.041	99
PR	44	0.151	0.127	991.58	0.491	0.477	90.3
GBT	2	0.139	0.130	153,521	0.452	0.486	99.9
RF	20	0.018	0.011	14.95	0.059	0.04	99.9
DT	1	0.019	0.01	7.79	0.061	0.039	99.9
NN	13	0.017	0.012	62.29	0.055	0.046	99.9
DL	18	0.020	0.012	17.17	0.065	0.045	99.9
K-NN	6	0.017	0.012	62.26	0.055	0.046	99.9
SVM	5	0.059	0.043	13.58	0.055	0.045	99.9

**Fig. 10:**
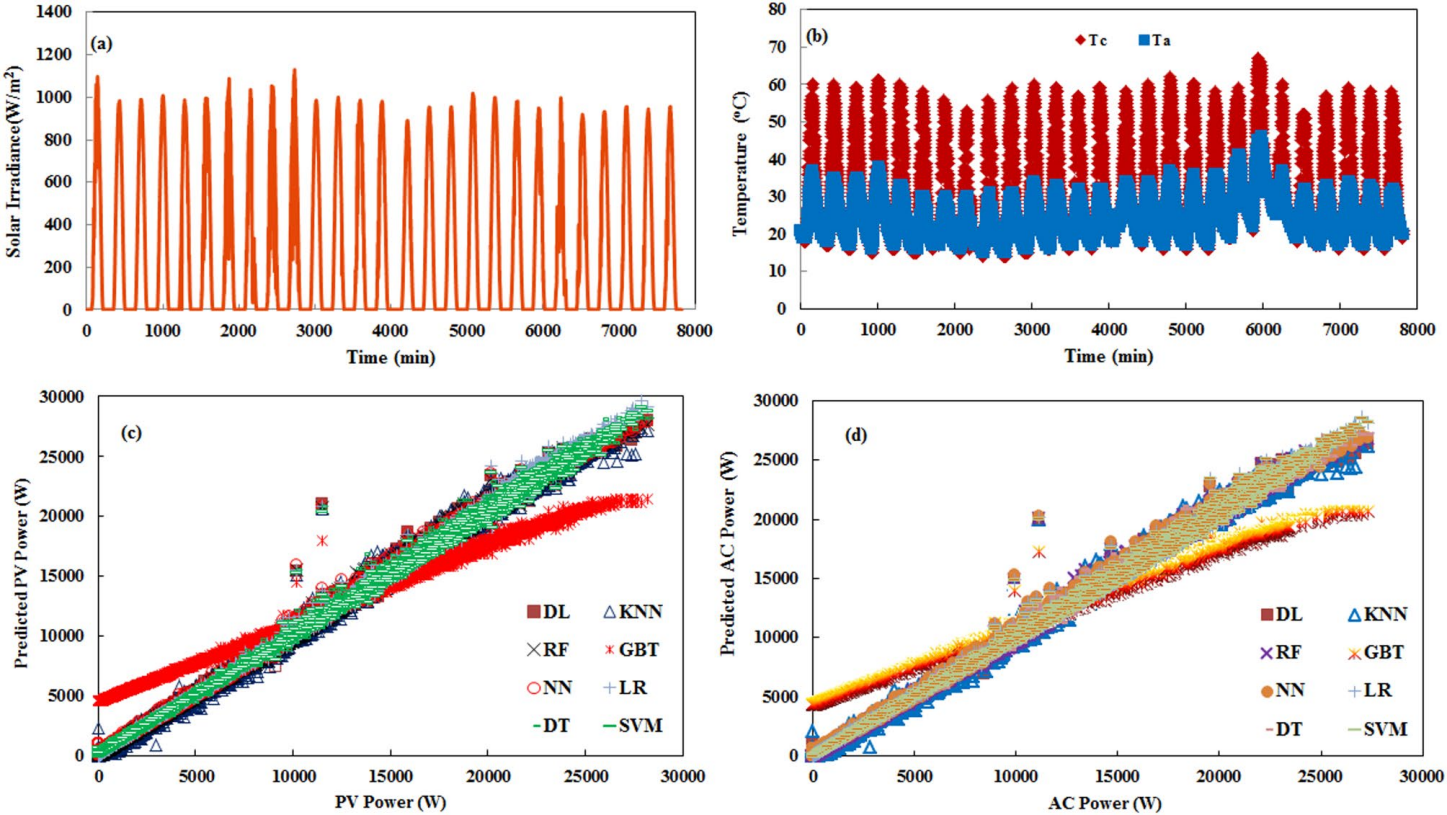
Long-term power forecasting for a month (**a**) solar radiation, (**b**) Temperature (cell and air), (**c**) PV power variation, and (**d**) AC power variation.

To evaluate model performance under long-term forecasting, the models were validated using a dataset spanning one full month of generation records. Figure 10 shows measured versus predicted PV output for April 2024, consisting of 7823 samples. Table 8 provides the analysis of machine learning algorithms for one-month PV power forecasting, revealing that DT delivers excellent performance, achieving the lowest RE (5.75%) while requiring minimal computational time (1 s). This outstanding efficiency-accuracy balance makes DT particularly valuable for operational long-term forecasting systems. RF and DL also demonstrate excellent performance with identical absolute errors (0.008) and low RMSE values (0.014), though RF achieves perfect correlation (*R* = 100%) at the cost of substantially higher computational demands (20 s). SVM shows notable improvement in monthly forecasting with competitive relative error (7.87%), but its extreme computational cost (193 s) limits practical application. NN and K-NN maintain strong performance with slightly higher error metrics, while LR offers reasonable accuracy with excellent computational efficiency. GBT and PR perform extremely poorly with relative errors exceeding 1,500%, confirming their unsuitability for PV forecasting. Most algorithms successfully capture the seasonal patterns in monthly PV generation (*R* ≥ 99.9%), but the significant variations in relative error despite similar correlation values highlight the importance of comprehensive performance evaluation. DT is the optimal choice for utilities and system operators requiring monthly PV forecasts for long-term planning and grid management, providing reliable predictions with minimal computational overhead while maintaining consistent excellence across multiple time horizons and weather conditions.

**Table 8:** The evaluation indices of the mlas models for a one-month time horizon.

MLA	Execution time (sec)	RMSE	AE	RE (%)	RRSE	NAE	*R* (%)
LR	1	0.010	0.036	21.96	0.057	0.036	99.9
PR	41	0.315	0.142	1,593.47	1.004	0.0513	70.1
GBT	3	0.133	0.124	1,554.53	0.424	0.449	99.9
RF	20	0.014	0.008	12.11	0.045	0.028	100
DT	1	0.015	0.008	5.75	0.048	0.027	99.9
NN	5	0.016	0.010	56.02	0.050	0.037	100
DL	16	0.014	0.008	21.13	0.043	0.028	99.9
K-NN	41	0.016	0.010	39.62	0.052	0.036	99.9
SVM	193	0.055	0.038	7.87	0.05	0.039	99.9

## Conclusion

This paper developed multi-label forecasting models for predicting the PV array and AC power outputs of a BAPV plant, utilizing different machine-learning algorithms and meteorological variables for short-, medium-, and long-term forecasts. The models were trained on a high-frequency dataset spanning one year of actual performance data, implemented and tested in the Rapidminer environment, and validated through experiments conducted at the ERI rooftop in Cairo, Egypt.

Key findings indicate that solar irradiance and ambient temperature significantly influence PV and AC power outputs. RF, DT, and DL models consistently demonstrated superior forecasting accuracy across all cases, achieving high correlation (*R* ≈ 99.8–100%) and low errors. For short-term forecasting, RF and NN performed best on sunny days, with RMSE as low as 0.023 and AE around 0.015, while DL, DT, and RF handled cloudy conditions well with RMSE ≈ 0.018 and AE ≈ 0.009. During sudden irradiance changes, RF, NN, and DL maintained high accuracy (*R* ≈ 99.8%) and minimal errors (RMSE ≈ 0.017–0.019). Medium-term forecasts over a week reinforced RF and DT as top models, achieving RMSE between 0.017 and 0.020, while K-NN and DL also performed well with RMSE ≈ 0.019–0.023. Long-term predictions over a month confirmed RF and DL’s dominance, with RMSE ≈ 0.014–0.018 and AE ≈ 0.008–0.012, ensuring stable and accurate forecasting. PR and GBT repeatedly exhibited extreme instability, with RMSE reaching 0.315 and relative errors exceeding 1,500%, making them unreliable for precise PV power forecasting. SVM demonstrated a strong correlation (99.9%) but suffered from excessive computation time (up to 193 s), reducing its practicality. DT remained the fastest model (1 s) while RF balanced accuracy and efficiency. Overall, RF, DT, and DL emerged as the best-performing models, offering the highest reliability, adaptability, and accuracy across different forecasting time horizons and weather conditions. The results across all time horizons demonstrate that the proposed models can accurately predict PV output from unseen measured meteorological data. The high correlation achieved, therefore, reflects the models’ strong capability to map known weather conditions to PV output, confirming their robustness and generalization ability under real-world conditions. The accurate forecasts produced can assist grid operators in anticipating variability in PV power output, thereby facilitating the integration of intermittent solar energy into the grid. Understanding how PV generation fluctuates under varying meteorological conditions is crucial for ensuring consistent integration of this weather-dependent power source. Furthermore, the multi-label predictions for both DC and AC power support inverter efficiency optimization and grid integration analysis, offering valuable insights for industry practitioners.

Future work should focus on incorporating additional variables and real-time data to enhance model robustness. This approach could lead to the creation of a generalized model capable of performing effectively under various weather conditions, thereby improving the reliability and efficiency of solar power integration into the grid.

## Data Availability

The datasets used and/or analysed during the current study are available from the corresponding author on reasonable request.

## Abbreviations

AE: Absolute error
AI: Artificial intelligence
BAPV: Building-applied photovoltaic
DL: Deep learning
DT: Decision trees
ET: Extra trees
GBT: Gradient-boosted trees
k-NN: K-nearest neighbor
LSTM: Long-short-term memory
L: Total number of layers in the deep neural network
LR: Linear regression
MAE: Mean absolute error
MAPE: Mean absolute percentage error
MASE: Mean absolute scaled error
ML: Machine learning
MLAs: Machine learning algorithms
MLP: A multilayer perceptron
NAE: Normalized absolute error
NN: Neural networks
PAC: AC power fed to the grid
PPV: PV array output power
PR: Polynomial regression
R: Correlation coefficient
RE: Relative error
RF: Random forests
RMSE: Root mean square error
RRSE: Relative root square error
Srad-W/m2: Solar irradiance
STC: Standard test conditions
SVM: Support vector machines
T: The total number of dts in the RF
Tamb-oC: Ambient temperature
Tcell-oC: Cell temperature
Ws-m/s: Wind speed
${b}_{j}^{\left(1\right)},\:{b}^{\left(2\right)}$: The biases for the hidden layer and output layer
$${\beta}_{0}$$: Model intercept
$${\beta}_{1},{\beta}_{2},{\beta}_{3},{\beta}_{n}$$: Predictors coefficients
${{c}_{i}}^{\left(t\right)}$: The constant value predicted by the t^th^ tree
$$\epsilon$$: Error term
${f}_{\text{h\:}}$, ${f}_{\text{out\:}}$: The activation functions for the hidden layer and the output layer
$\mu$: The mean of the data, and *σi* is the standard deviation of the data
${\upomega}$: Weight matrix for layer
${x}_{i}$, $\bar{x}$: The values and the mean of the x-variable in a dataset
${y}_{i}$, $\bar{y}$: The values and the mean of the y-variable in a dataset
$\widehat{zs}$: The scaled value, $zi$ is the measured value
${z}_{max}$, ${z}_{min}$: The maximum and the minimum values of the dataset
